# ADAR expression and copy number variation in patients with advanced gastric cancer

**DOI:** 10.1186/s12876-020-01299-8

**Published:** 2020-05-14

**Authors:** Javad Behroozi, Shirin Shahbazi, Mohammad Reza Bakhtiarizadeh, Habibollah Mahmoodzadeh

**Affiliations:** 1 Department of Medical Genetics, Faculty of Medical Sciences, Tarbiat Modares University, Tehran, Iran; 2 Department of Animal and Poultry Science, College of Aburaihan, University of Tehran, Tehran, Iran; 3 Department of Surgical Oncology, Cancer Institute, Imam Khomeini Hospital Complex, Tehran University of Medical Sciences, Tehran, Iran

**Keywords:** Gastric cancer, ADAR gene, Overexpression, Amplification, Prognosis

## Abstract

**Background:**

Gastric cancer (GC) is a world health problem and it is the third leading cause of cancer deaths worldwide. The current practice for prognosis assessment in GC is based on radiological and pathological criteria and they may not result in an accurate prognosis. The aim of this study is to evaluate expression and copy number variation of the ADAR gene in advanced GC and clarify its correlation with survival and histopathological characteristics.

**Methods:**

Forty two patients with stage III and IV GC were included in this study. ADAR gene expression and copy number variation were measured by real-time PCR and Quantitative multiplex fluorescent-PCR, respectively. Survival analysis performed based on the Kaplan–Meier method and Mantel–Cox test.

**Results:**

ADAR mRNA was significantly overexpressed in the tumor tissues when compared to the adjacent normal tissues (*p* < 0.01). Also, ADAR expression level in stage IV was higher than stage III. 40% of patients showed amplification in ADAR gene and there was a positive correlation between ADAR copy number and expression. Increased ADAR expression was clearly correlated with poorer survival outcomes and Mantel–Cox test showed statistically significant differences between low and high expression groups (*p* < 0.0001). ADAR overexpression and amplification were significantly associated with metastasis, size and stage of tumor.

**Conclusions:**

Together, our data indicate that amplification leads to over expression of ADAR and it could be used as a prognostic biomarker for disease progression, especially for the metastatic process in GC.

## Background

Gastric cancer (GC) currently ranks fourth in cancer incidence worldwide. Gastric adenocarcinoma is the most common histological type of all malignancies originating in the stomach and known as a heterogeneous disease with diverse phenotypes and genotypes. Although in the last few decades its prevalence has declined to reach a plateau, GC still is the third leading cause of cancer deaths worldwide [1]. Surgery is the most helpful and reliable procedure to eradicate the disease, especially in the case of primary tumors. Nevertheless, the results for advanced GC remain unfavorable even when extensive surgery had been performed [2]. Studies from several groups over the past decade have now produced a near-comprehensive catalogue of genetic alterations in GC [3]. Certain genetic and epigenetic molecular abnormalities such as tumor suppressor gene mutations, gene changes inducing loss of normal cellular adhesion, over-expression or mutations of cell membrane receptors with tyrosine kinase activity or activation of angiogenic factors have been reported to be involved in the pathogenesis and progression of GS [4, 5].

RNA editing is an important form of post-transcriptional processing, which can alter RNA molecules by deamination of adenosine to form inosine. This reaction is catalyzed by a family of enzymes named adenosine deaminase acting on RNA (ADARs) [6]. The human genome contains three ADAR genes including ADAR, ADARB1 and ADARB2. The first two are ubiquitously expressed and catalytically active, but, ADARB2 exclusively expressed in the brain and has not shown any catalytic activity so far [7]. Although both ADAR and ADARB1 have been shown to play roles in tumorigenesis, most of cancer related editing events regulated by ADAR, primarily due to more abundant expression of ADAR and its unique features [8].

It is estimated that human transcriptome undergoes over 100 million editing events, which may result in codon changes with the consequence of altered protein function, alternative splicing or affect targeting and maturation of microRNAs [9]. ADAR with the ability to change DNA-encoded genetic information after transcription, could be an important contributor in cancer development [10]. Hence, the inconsistency in ADAR expression or activity may be a causative factor in a variety of diseases including cancer [11]. Amplification and overexpression of the ADAR gene occurs in over 8% of breast, lung, and hepatic cancers [12].

Acute myeloid leukemia was the first cancer that altered mRNA editing was shown to be connected to the disease [8]. Thereafter, ongoing studies have been elucidate role of ADAR in cancer development and progression. It has been shown that increased ADAR expression correlates with tumor recurrence in lung adenocarcinoma [13]. In addition, ADAR overexpression is connected with increased malignancy of breast, lung and liver cancer, and silencing of ADAR in breast cancer cells results in increased apoptosis. The latter suggests that ADAR has anti-apoptotic function that promotes cancer progression [14].

Although significant progress has been made in the management and treatment of GC patients, however, further studies should be conducted to promises discoveries of new biomarkers and more innovative and effective treatments for patients with GC. The main end point of our study is to evaluate expression and copy number variation of ADAR (ADAR1) gene in advanced GC and clarify its correlation with overall survival and histopathological characteristics.

## Methods

### Patients and specimen collection

Fresh frozen tumor and normal adjacent tissue of 42 patients with stage III and IV gastric adenocarcinoma who underwent surgery at the Cancer Institute of Iran were examined in this study. We included only GC patients that did not receive neoadjuvant therapy. The biological materials were provided by the Iran National Tumor Bank, founded by the Cancer Institute of Tehran University of Medical Sciences, for cancer research. Staging were based on the American Joint Committee on Cancer (AJCC) cancer staging manual [15]. All samples were transferred in liquid nitrogen from Tumor bank and stored at − 80 °C for further investigations. Complete clinicopathologic data including patient history, histology, clinical, and paraclinical data and follow-up information were gathered from the medical records of every patient. All participants signed written informed consent.

### RNA/DNA extraction

RNA and DNA were purified from normal and tumoral samples using the All-in-one DNA/RNA/Protein Mini-Preps Kit (Biobasic, Canada) following manufacturer’s protocol. The RNA quality was verified by electrophoresis on 1.0% agarose gel. Also, the quantity of isolated RNA and DNA was evaluated by NanoPhotometer (NP80, Germany).

### cDNA synthesis and real-time PCR

RNA samples were reverse transcribed into complementary deoxyribonucleic acid (cDNA) using Easy™ cDNA Synthesis Kit (Parstous biotechnology, Iran). The cDNA was synthesized from 1 μg of total RNA with random hexamer and oligo (dT) as reaction primers in a final volume of 20 μl following manufacturer’s protocol.

Obtained cDNA was further amplified by real-time quantitative PCR using RealQ Plus Master Mix Green (Ampliqon, Denmark) based on the kit protocol. The primers used for performing real-time PCR were as follows: for ADAR1 F: 5′AGCTTGGGAACAGGGAATCG3′ and R: 5′CTTCGCAGTCTGGGAGTTGT3′; for GAPDH F: 5′ACACCCACTCCTCCACCTTTG3′ and R: 5′TCCACCACCCTGTTGCTGTAG3′. Real-time PCR mixture contained 10 μl of SYBR green master mix, 1 μl of forward and reverse primers and 2 μl of cDNA. Real-time PCR program for the reaction was based on a holding step at 95 °C for 15 min, followed by 40 cycles of denaturing at 95 °C for 20s, annealing and extending at 60 °C for 40s. Real-time PCR was performed at least three times in the StepOne™ Real-Time PCR System (Applied Biosystems, USA) for each sample and fold change of gene expression was calculated using 2^-∆∆Ct^ method.

### Quantitative multiplex fluorescent-PCR (QMF-PCR)

The ADAR gene copy number was quantified by QMF-PCR on a BIOER XP Cycler (Bioer, China). The QMF-PCR technique consists of the quantification of fluorescently labeled test and control amplicons, obtained by a single multiplex PCR amplification. Primers were designed for 5′UTR, exonic and 3′UTR of the ADAR gene. Three other genes including BOD1L, AGBL2 and POR were co-amplified as controls. A tail of universal primer was added to forward primers, except for POR gene which the tail was added to reverse primer. The universal primer was labeled with the fluorescent phosphoramidite 6-FAM dye and all the primers were HPLC purified. Multiplex PCR employed the Multiplex TEMPase Master Mix (Ampliqon, Denmark) following the manufacturers recommendations. Primers stock solution was prepared in a 0.5:1:0.5 ratio of forward primer, reverse primer and fluorescently tagged universal primer, respectively. The reaction started with an initial denaturation of 15 min at 95 °C, followed by 30 cycles at 95 °C for 45 s, 59.8 °C for 45 s and 72 °C for 45 s, and a final extension of 15 min at 72 °C. Primer sequences used for QMF-PCR are shown in Table 1.

**Table 1:** List of primer sequences used for QMF-PCR analysis in this study. Universal tail was shown in bold letters

Gene	Primer Sequence	
ADAR-3′UTR	F	**GCCTCCCTCGCGCCA**TAGACTTGGTGCCGTGGTGA
	R	GTCGCAGAGCCTCAGTAGTC
ADAR-Exonic	F	**GCCTCCCTCGCGCCA**TCGACTTGTAACCGGCCTGA
	R	GTTGTAAACGAACCCAGACGG
ADAR-5′UTR	F	**GCCTCCCTCGCGCCA**GGGGACCACTTACAAGCTGATG
	R	GTCTGGTCGCAGATTGGTGA
AGBL2	F	**GCCTCCCTCGCGCCA**GCGAGCTGCATTCCATGCG
	R	TCCCAGCTTTGGAAACGCAC
BOD1L	F	**GCCTCCCTCGCGCCA**AATGCCTCCGCTTTCAGGC
	R	ATCACTTGGCAACTCACACATGG
POR	F	AGCCACTTTGTGCCAGATCA
	R	**GCCTCCCTCGCGCCA**TCCAGCACGTGTTCACATCA
Universal	U	FAM-GCCTCCCTCGCGCCA

One μl of the PCR products was added to 10 μl of formamide and 0.5 μl of GeneScan-500 LIZ size standard (Applied Biosystems, USA). Then, the PCR product was denatured at 95 °C for 3 min and placed on ice to prevent re-annealing until further analysis. Fragment analyses were performed using a POP7 gel on the ABI 3500 Genetic Analyzer (Applied Biosystems, USA). The results were processed with GeneMarker® software V2.7 (SoftGenetics, USA) to obtain electropherograms for each sample. Each product was identified by its size, and the area under peak were imported into an Excel spreadsheet and the copy number of each amplicon was determined by calculating a dosage quotient (DQ) for each fragment [16, 17].

### Cell lines ADAR gene expression and CNV

Biological databases are crucial for exploring the molecular mechanisms of cancer, therefore, basal expression of ADAR mRNA and ADAR copy number in 37 GC cell lines were obtained from cancer cell line Encyclopedia (CCLE) [18]. RNA expression values were reported in reads per kilobase of transcript per million mapped reads (RPKM). Finally, the association of the RNA expression values and gene copy numbers for each cell line was examined.

### Survival analyses

To investigate the relationship between ADAR expression and prognosis of gastric cancer, survival data associated with GC patients were obtained from Kaplan-Meier Plotter [19]. Overall survival (OS) data for 876 patient (312 low expression and 564 high expression) were analyzed to create Kaplan-Meier plots. These patients were related to GSE14210, GSE15459, GSE22377, GSE29272, GSE51105 and GSE62254 datasets.

### Statistical analyses

All statistical analyses were performed using SPSS 18.0 (SPSS Inc., USA) and GraphPad Prism 8 (GraphPad Software, USA) software. Paired T-test was applied in comparisons of ADAR mRNA levels between GC tissue samples and their paired non-tumor counterparts and unpaired T-test was used for comparison between stage III and IV. The Chi-square test was performed to evaluate correlations between ADAR expression or amplification and clinicopathological parameters. Pearson’s correlation coefficient was used to determine the relationship between the expression and CNV of ADAR in GC cell lines. Differences in the OS between the high and low expression groups were estimated and compared by the Kaplan–Meier method with a Mantel–Cox test. Differences were considered significant when the *P*-value was < 0.05.

## Results

### ADAR gene expression in gastric cancer tissue

**Fig. 1:**
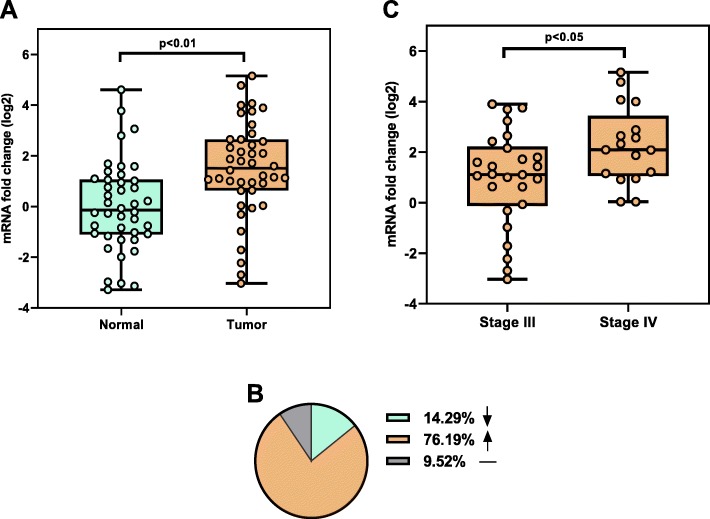
ADAR gene expression in GC patients (**a**) matched normal and tumor tissues, (**b**) percent of ADAR dysregulation, (**c)** stage IIIC compared with Stage IV

Among the 42 tumor specimens tested, average expression of ADAR mRNA was significantly upregulated in the tumor tissues when compared to the adjacent normal tissues of the GS patients (*p* < 0.01, Fig. 1). In tumor samples ADAR was expressed 2.8-fold higher than normal tissues. That is to say, the level of ADAR mRNA expression was increased in 32 (76.2.0%) tumors while it was decreased in 6 (14.3%) cases and it has remained unchanged in 4 (9.5%) tumors (Fig. 1b). The average expression of ADAR mRNA was lower in the stage III in comparison to stage IV (*p* = 0.0225, Fig. 1c).

### ADAR copy number variation

**Fig. 2:**
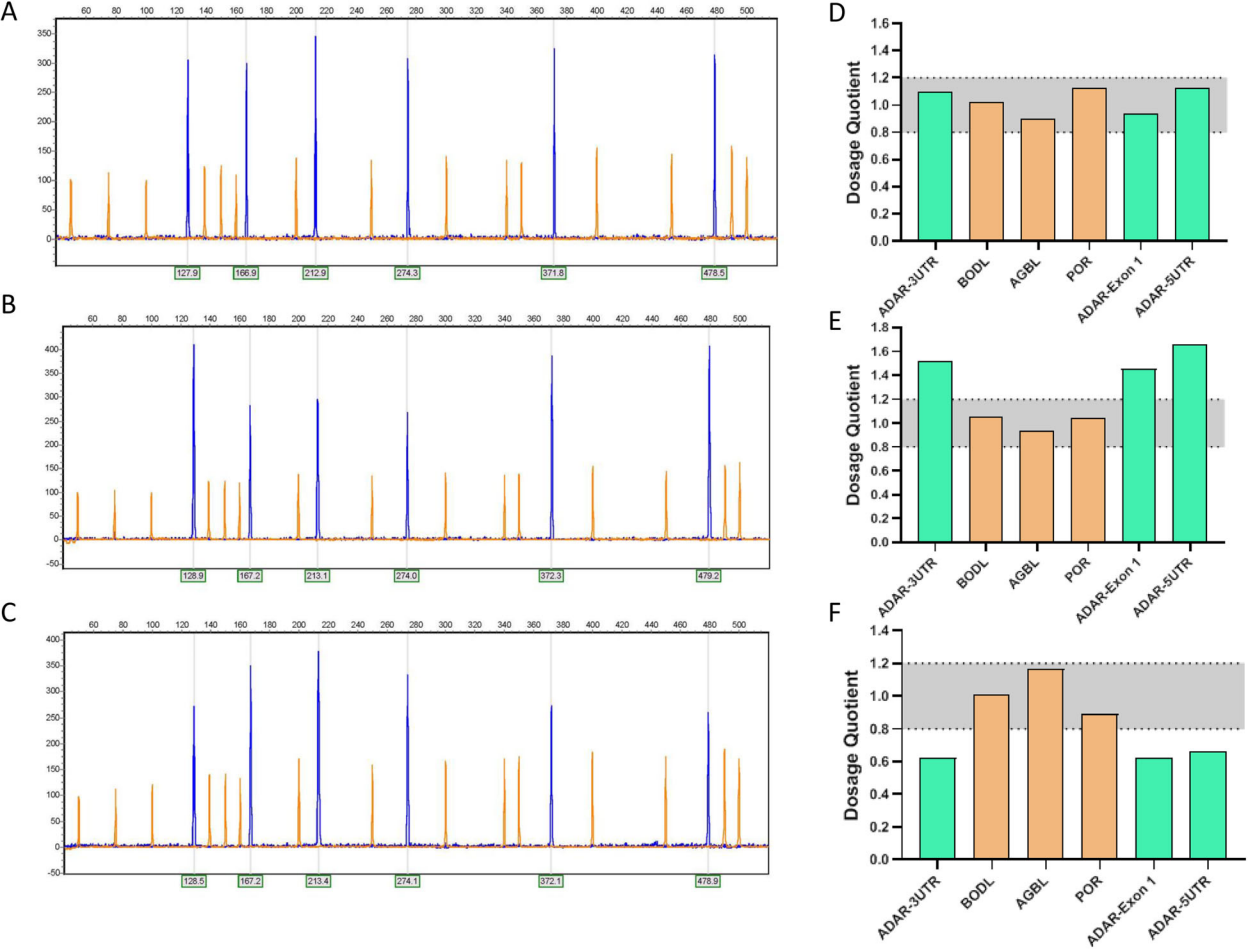
Electropherogram and dosage quotient of a sample with (**a**) and (**d**) diploid copy number, (**b**) and (**e**) amplification, (**c**) and (**f**) deletion

**Fig. 3:**
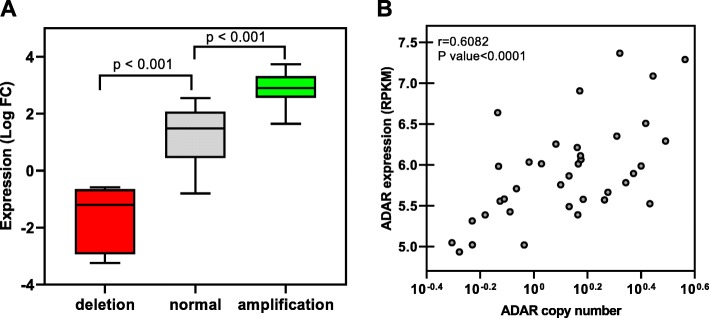
Correlation of CNV and gene expression in GC patients (**a**) patients and (**b**) GC cell lines

According to DQ of the three segments of ADAR gene, we divided ADAR CNV into deleted, amplified and diploid categories (Fig. 2). Our results showed 20 (40%) tumors with diploid copy number, 5 (12%) tumors with deleted and 17 (40%) tumors with amplified ADAR gene. Patients with amplification showed significant increase in ADAR gene expression compared with patients with diploid and deleted ADAR copy number (Fig. 3a). Analysis of ADAR copy number in GC cell line were consistent with tumor specimens. We found a positive correlation (r = 0.61, *p* < 0.0001) between ADAR copy number and expression (Fig. 3b).

### Correlations between ADAR expression and CNV with clinicopathological features

Here, we analyzed the association of the ADAR gene CNV and its expression with the clinicopathological features of patients. Forty two tissue samples based on ADAR gene expression were classified into 2 groups: high expression (FC > 4.56, *n* = 17) and low expression (FC ≤4.56, *n* = 25). This study consisted of 25 patients aged ≥66 years and 17 patients aged < 66 years (*P* = 0.573). ADAR high expression was significantly associated with metastasis (*p* = 0.008), stage (*p* = 0.008), histology (*p* = 0.015) and size of tumor (*p* = 0.004). Moreover, amplification was significantly associated with metastasis (*p* < 0.018), stage (p < 0.018) and size of tumor (*p* = 0.008) in GC patients and tended to be associated with histology (*p* = 0.143). Furthermore, no significant association was detected between ADAR gene CNV and its expression and other clinicopathological parameters, including age, gender, regional lymph node metastases, site of primary tumor and Lymphovascular invasion (Table 2).

**Table 2:** Correlation between clinicopathological variables and ADAR expression and CNV in Gastric cancer patients

variable	ADAR expression		***P*** value	ADAR amplification		***P*** value
	High(17)	Low(25)		Yes(18)	No(24)	
**Age (year)**						
≥ 66^a^	11	14	0.573	11	14	0.856
< 66	6	11		7	10	
**Gender**						
Male	11	15	0.758	12	14	0.582
Female	6	10		6	10	
**Pathological N category**						
N0	1	1	0.074	1	1	0.086
N1	1	4		0	5	
N2	5	15		8	12	
N3	10	5		9	6	
**M classification**						
M0	6	19	0.008^*^	7	18	0.018^*^
M1	11	6		11	6	
**Site of primary tumor**						
Antrum	4	6	0.937	4	6	0.315
Body	8	13		9	12	
Cardia	4	4		5	3	
Fundus	1	2		0	3	
**Histology**						
Well differentiated	0	5	0.015^*^	1	4	0.143
Moderately differentiated	3	11		4	10	
Poorly differentiated	10	8		9	9	
Undifferentiated	4	1		4	1	
**Size of primary tumor**						
< 6.1	7	21	0.004^*^	8	20	0.008^*^
≥ 6.1	10	4		10	4	
**Tumor stage**						
Stage 4	11	6	0.008^*^	11	6	0.018^*^
Stage 3	6	19		7	18	
**Lymphovascular invasion**						
Present	14	16	0.196	15	15	0.139
Absent	3	9		3	9	

* indicates *p*-value < 0.05. ^a^ the median age at surgery was 66 years

### Prognostic value of ADAR in gastric cancer

**Fig. 4:**
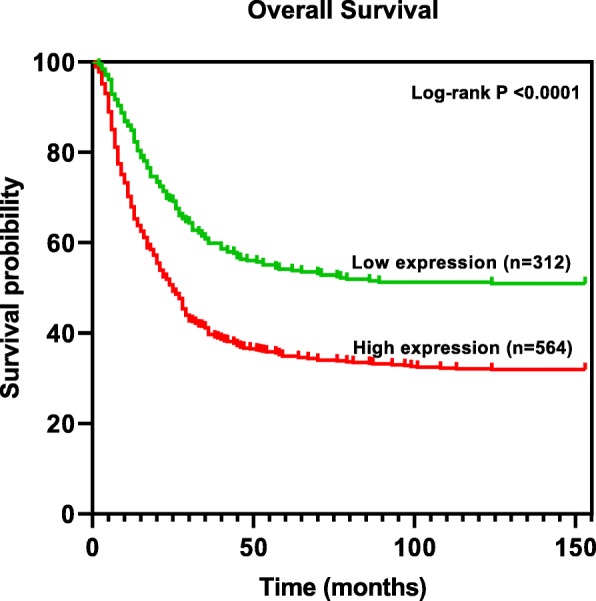
Kaplan-Meier survival curves according to high and low expression of ADAR

The GC database used in this study, includes 876 samples from six independent datasets. Kaplan–Meier analysis of this dataset indicated that increased ADAR expression was clearly correlated with poorer survival outcomes (Fig. 4). Patients in the high expression group had a significantly shorter OS than patients in the low expression group (log-rank test, *P* < 0.0001). Median survival of high and low expression groups were 23.2 and 70.2 months, respectively. Furthermore, median survival of patients with different characteristics was obtained. Patients with high expression of ADAR have significantly lower median survival time in most subgroups (Table 3).

**Table 3:** Characteristics and median survival of patients in high and low expression groups

Characteristics	Median survival (months)		Log-rank P
	High expression	Low expression	
**Gender**			
Male (*n* = 544)	20.3	32.1	1.6e-5
Female (*n* = 236)	15.2	93.2	4.3e-7
**Tumor stage**			
Stage 1 (*n* = 67)	NA^a^	NA	–
Stage 2 (*n* = 140)	29	78.6	0.103
Stage 3 (*n* = 305)	22.4	52.6	2e-4
Stage 4 (*n* = 148)	17.5	15.93	0.148
**Histology**			
Well differentiated (*n* = 32)	14.5	45.1	0.053
Moderately differentiated (*n* = 67)	30.4	56.9	0.428
Poorly differentiated (*n* = 165)	23.6	40	0.105
**Lauren classification**			
Intestinal (*n* = 320)	25.9	99.4	3.1e-5
Diffuse (*n* = 241)	27.8	40	0.038
Mixed (*n* = 32)	57.2	20.9	0.041
**Treatment**			
Surgery (*n* = 380)	45.8	85.6	0.217
5-Fluorouracil (*n* = 152)	9.2	21.3	3.3e-6

^a^ NA means no death in follow up threshold

## Discussion

Application of molecular biomarkers in clinical setting might improve diagnostic and management strategy of GC patients. To date, some molecular markers, including human epidermal growth factor receptor-2, vascular endothelial growth factor receptor 2, urothelial cancer associated 1, excision repair cross-complementation group 1, B-cell lymphoma-2, and Ki-67 have been proposed to have diagnostic and prognostic value in the management of GC patients [20, 21]. However, the majority of these markers were not able to accurately reflect prognostic value and therapeutic efficiency in advanced GC. Identification of molecular biomarkers might improve patient therapeutic strategy of GC, hence, we evaluated the CNV and expression of ADAR to reveal its alteration and clinical significance in advanced GC.

We analyzed mRNA expression level of ADAR in GC tumors and adjacent normal tissues. This revealed that ADAR is remarkably overexpressed in GC tumors. In addition, we compared ADAR expression level in GC stage III and IV. Interestingly, the average expression of ADAR mRNA in stage IV was higher than stage III. Besides, overexpression of ADAR was significantly associated with metastasis, stage, histology and size of tumor, which may indicate that ADAR has a pivotal role in stage III to IV progression of GC. According to ADAR function, overexpression of ADAR could leads to hyper-editing [22]. Unbalanced editing process has adverse results, and abnormal RNA editing within the transcriptome detected in many kinds of tumors. RNA editing could leads to recoding of a transcript and contribute to carcinogenesis through reducing the activity of tumor suppressors such as bladder cancer associated protein in hepatocellular carcinoma [23] or enhancing the activity of pro-survival genes such as antizyme inhibitor 1 in cervical cancer [24].

To determine whether ADAR copy number has variation in tumors, QMF-PCR was conducted. We found that ADAR significantly amplifies in advanced GC. Moreover, our results showed a positive correlation between ADAR gene copy number and its expression at the mRNA level. Correlation between ADAR copy number and ADAR expression has also been reported in breast carcinoma, ovarian adenocarcinoma, lung adenocarcinoma and liver carcinoma [24]. ADAR frequently amplifies in human cancers consistent with the elevated expression and editing levels of its substrates [25]. Notably, knockdown of ADAR in lung adenocarcinoma cells with amplified ADAR leads to decreased migration and invasion [13]. Hence, pharmacological targeting of ADAR promise a potential therapeutic application for tumors with ADAR amplification.

Also, we carried out a correlation test between ADAR expression and copy number change in 37 GC cell lines and statistical analysis revealed that ADAR expression was consistently associated with ADAR copy number. The association of gene copy number with gene expression has also been found in other cancer cell lines. Hyman et al. performed a high-resolution analysis in breast cancer cell lines and showed that 44% of the highly amplified genes showing overexpression and 10.5% of the highly overexpressed genes being amplified [26]. These findings further strengthen our hypothesis that ADAR amplification increases its expression.

As previously mentioned, ADAR amplification and overexpression in different cancers have been reported [24, 25]. Yet, the effects of these genomic and transcriptomic changes on clinicopathological features of GC patients have remained largely unknown. We evaluated the association of the ADAR gene CNV and its expression with the clinicopathological features of patients. The mRNA expression levels of ADAR were positively associated with metastasis, stage, histology and size of tumor. Indeed, ADAR amplification was significantly associated with metastasis, stage and size of tumor. Findings of the current study support the previous study reporting the oncogenic potential of ADAR in GC [27]. The association between the tumor aggressiveness and the overexpression of ADAR has also been demonstrated in hepatocellular carcinoma and colorectal cancer [28, 29]. Based on available evidences, the molecular mechanisms of how ADAR promotes GC cell growth and migration might be explained by its role in the regulation of mTOR signaling pathway. Overexpression of ADAR in GC cells increases proliferation and migration but these effects significantly debilitate with rapamycin, the mTOR kinase inhibitor, demonstrating that rapamycin could inhibit the effects of ADAR overexpression on GC cell growth and migration. Together these results suggested that mTOR signaling is important for ADAR mediated GC invasion and metastasis [27].

Currently, prognosis of GC patients is primarily determined using depth of wall invasion, lymph node or distant metastasis status and age; however, these prognostic factors are limited in clinical practice, and may not result in an accurate prognosis [30]. To further confirm the prognostic value of ADAR overexpression in GC patients, Kaplan–Meier analysis and log-rank test were performed. The results showed that the overall survival rate of patients with lower expression of ADAR was better than that of patients with high expression, suggesting its prognostic role. Chen et al. found a correlation between high expression of ADAR and a poor prognosis of cervical cancer [24]. Again, Chan et al. showed a significant correlation between ADAR upregulation and GC patient shorter survival [31].

## Conclusions

The current study demonstrates that ADAR mRNA is overexpressed in GC through DNA copy number amplification. ADAR overexpression and amplification correlate with the main negative clinicopathological factors such as metastasis, tumor size and stage in GC patients. Furthermore, increased ADAR expression was clearly correlated with poorer survival outcomes. Therefore, ADAR overexpression is not only a biomarker of tumor progression but also contributes to tumor progression. We propose ADAR as useful prognostic markers and therapeutic targets for GS. Doubtless, further investigations are necessary to confirm these primary results.

## Data Availability

The datasets used and/or analyzed during the current study are available from the corresponding authors upon reasonable request.

## Abbreviations

GC: Gastric cancer
ADAR: Adenosine deaminase acting on RNA
cDNA: Complementary deoxyribonucleic acid
QMF-PCR: Quantitative multiplex fluorescent-PCR
CNV: Copy number variation
OS: Overall survival
DQ: Dosage quotient
